# The architecture of *Trypanosoma brucei* tubulin-binding cofactor B and implications for function

**DOI:** 10.1111/febs.12308

**Published:** 2013-05-24

**Authors:** Jennifer R Fleming, Rachel E Morgan, Paul K Fyfe, Sharon M Kelly, William N Hunter

**Affiliations:** 1 Division of Biological Chemistry and Drug Discovery, College of Life Sciences, University of DundeeUK; 2 Institute of Molecular, Cell and Systems Biology, College of Medical, Veterinary and Life Sciences, University of GlasgowUK

**Keywords:** CAP-Gly domain, CD, crystallography, tubulin-binding, ubiquitin-like

## Abstract

Tubulin-binding cofactor (TBC)-B is implicated in the presentation of α-tubulin ready to polymerize, and at the correct levels to form microtubules. Bioinformatics analyses, including secondary structure prediction, CD, and crystallography, were combined to characterize the molecular architecture of *Trypanosoma brucei* TBC-B. An efficient recombinant expression system was prepared, material-purified, and characterized by CD. Extensive crystallization screening, allied with the use of limited proteolysis, led to structures of the N-terminal ubiquitin-like and C-terminal cytoskeleton-associated protein with glycine-rich segment domains at 2.35-Å and 1.6-Å resolution, respectively. These are compact globular domains that appear to be linked by a flexible segment. The ubiquitin-like domain contains two lysines that are spatially conserved with residues known to participate in ubiquitinylation, and so may represent a module that, through covalent attachment, regulates the signalling and/or protein degradation associated with the control of microtubule assembly, catastrophe, or function. The TBC-B C-terminal cytoskeleton-associated protein with glycine-rich segment domain, a known tubulin-binding structure, is the only such domain encoded by the *T. brucei* genome. Interestingly, in the crystal structure, the peptide-binding groove of this domain forms intermolecular contacts with the C-terminus of a symmetry-related molecule, an association that may mimic interactions with the C-terminus of α-tubulin or other physiologically relevant partners. The interaction of TBC-B with the α-tubulin C-terminus may, in particular, protect from post-translational modifications, or simply assist in the shepherding of the protein into polymerization.

## Introduction

Key to the regulation of many biological processes is the tight control exercised over protein biosynthesis, folding, and degradation. With respect to tubulin, a central component of the cytoskeleton, the correct folding and polymerization involves distinct stages that are influenced by several chaperones or cofactors 1–2. Initially, after a tubulin polypeptide is produced, it is captured by prefoldin 3 and subsequently passed to chaperonin-containing T-complex polypeptide 1 (CCT) 4. When released from CCT, tubulin is essentially folded, but appears to be unable to polymerize and form microtubules (MTs) 1. Our understanding of what occurs between release from CCT and MT formation is limited. Five tubulin-binding cofactors (TBCs) are implicated in late-stage tubulin folding and heterodimer assembly 1,5–10. TBC-B and TBC-E are implicated in binding α-tubulin, whereas TBC-A and TBC-D interact with β-tubulin. TBC-C is involved in the final stages of dimer formation, stimulating GTP hydrolysis in β-tubulin and heterodimer release from a protein assembly 2. Little is known about the molecular basis for the roles of these cofactors, a point that we sought to address in relation to TBC-B.

**Fig 1:**
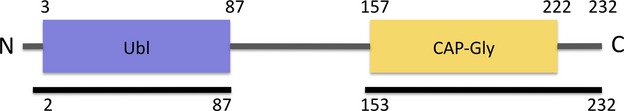
Schematic of *Tb*TBC-B. Domains and termini are labelled. Residues 3–87 and 157–222 represent the assigned Ubl (blue box) and CAP-Gly (yellow box) domains, respectively. Black lines and residue numbers 2–87 and 153–232 represent the structures determined.

TBC-B comprises an N-terminal ubiquitin-like (Ubl) domain and a C-terminal cytoskeleton-associated protein with glycine-rich segment (CAP-Gly) domain. There are NMR structures of the TBC-B Ubl domains from *Caenorhabditis elegans* 11, *Drosophila melanogaster* [Protein Data bank (PDB) code 2KJR], *Arabidopsis thaliana* (2KJ6), and *Mus musculus* (1V6E). The mouse TBC-B CAP-Gly domain NMR structure has also been determined (1WHG), but the only crystal structure known is that of the *C. elegans* TBC-B CAP-Gly domain 12. We targeted the protein from *Trypanosoma brucei*, an organism that is considered to be a useful model for the study of MT biology 13. Searching against the translated *T. brucei* genome (http://www.genedb.org/) with mouse, human and *C. elegans* TBC-B sequences identified a single protein, *Tb*10.61.2930, with ∼ 40% amino acid sequence identity. This protein, *T. brucei* TBC-B (*Tb*TBC-B), consists of 232 amino acids organized into the N-terminal Ubl domain (∼ 90 residues) and a CAP-Gly domain (residues 157–222; Fig. 1).

We now report the construction of an efficient bacterial recombinant expression system, protein purification and the use of CD and bioinformatics approaches to investigate secondary structure content and predicted flexibility. Crystallization of the full-length protein was achieved, but the samples were poorly ordered. Consequently, structural analyses of the individual domains were carried out. The fortuitous observation in the crystal structure of the CAP-Gly domain of intermolecular contacts with the C-terminus of a symmetry-related molecule suggests how interactions with partners, including α-tubulin, might occur. Comparisons with ubiquitin suggest a functional link between the structure of TBC-B and the regulation of distinct populations of proteins associated with tubulin biology by proteasome-dependent degradation.

## Results and Discussion

Recombinant full-length *Tb*TBC-B was produced in *Escherichia coli* and purified. The final step in purification was size exclusion gel chromatography, and this indicated that the full-length protein is monomeric in solution. The protein crystallized readily; however, despite displaying a good appearance (data not shown), X-ray diffraction from the crystals did not extend beyond 7 Å of resolution. Limited proteolysis produced two polypeptides, one of which, the Ubl domain, gave highly ordered crystals, and the structure was solved with anomalous dispersion methods 14. Subsequently, crystals of the CAP-Gly domain were also obtained after testing of a series of truncated constructs and molecular replacement allowed for structure solution. We note, that following proteolysis, the two domains were readily separated and purified, indicating that there was no domain–domain association, and when studied individually they remained monomeric in solution.

### The Ubl domain

The N-terminal Ubl domain of *Tb*TBC-B crystallized in the tetragonal space group *P*4_1_2_1_2 with a single polypeptide in the asymmetric unit. Size exclusion chromatography employed during purification indicated that the protein was monomeric in solution.

**Fig 2:**
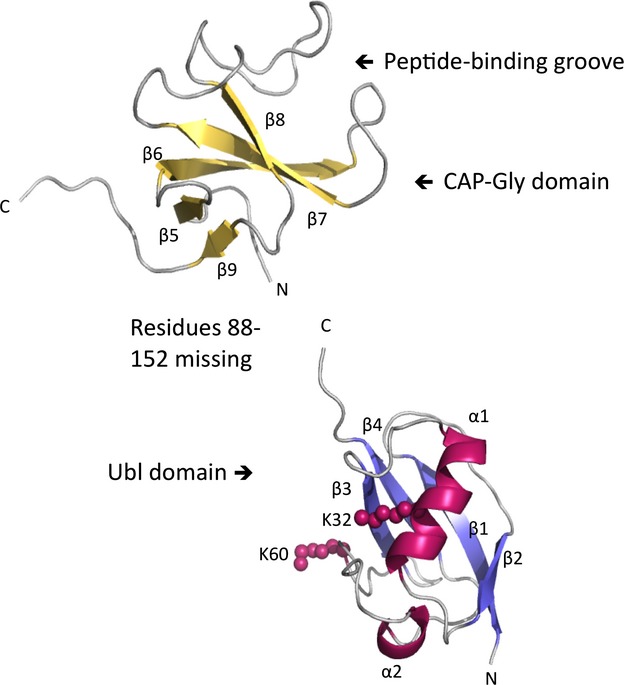
Ribbon diagram of the two domains of *Tb*TBC-B. The Ubl domain β-strands are in light blue, α-helices are in pink, and the CAP-Gly domain β-strands are in yellow. The N-termini and C-termini and elements of secondary structure are labelled. The orientation of the two domains with respect to each other is arbitrary. Two lysines of note are depicted as pink spheres.

The Ubl domain is a small globular entity consisting of a mixed four-strand β-sheet that forms a concave groove in which a single α-helix is placed (Fig. 2). This is an example of a β-grasp fold, a common structure involved in protein–protein interactions 5–11. A pronounced hydrophobic core is present, formed mainly by aliphatic side chains. The residues involved, which have < 10% solvent-accessible surface area, include Val4, Val6, Leu8, Tyr22, Ile28, Ile31, Val35, Thr41, Met46, Leu48, Leu50, Met62, Leu68, Cys73, Ile79, and Val81 (not shown). These core residues are, in general, conserved among the TBC-B family of proteins. The surface residues are more variable, perhaps indicative of a module that does not interact directly with α-tubulin. Note that, as α-tubulin is a very highly conserved protein, any interacting components would be expected to display conservation on their surfaces if they interacted at similar positions.

**Fig 3:**
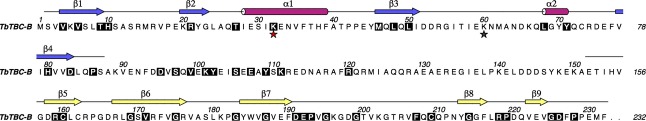
The primary and secondary structure of *Tb*TBC-B. Residues strictly conserved in at least 75% of TBC-B sequences are encased in black. Red stars mark Lys32 and Lys60, which are conserved in ubiquitin.

**Fig 4:**
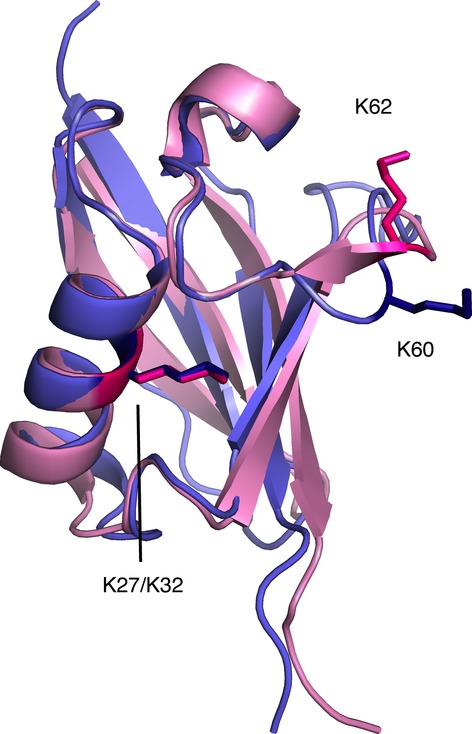
Structural overlay of *Tb*TBC-B Ubl (blue) and ubiquitin (PDB code 1UBQ; pink). Spatially conserved lysines are depicted as sticks.

**Fig 5:**
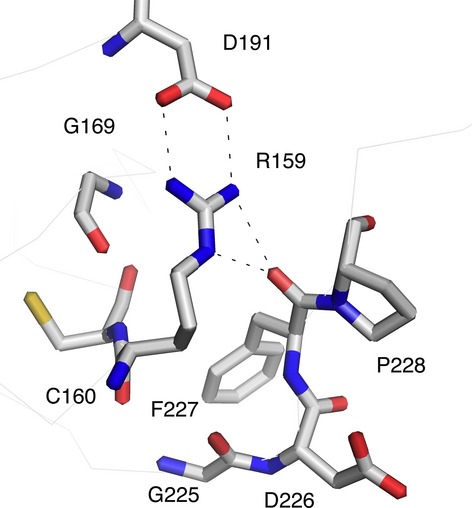
(A) The *Tb*TBC-B Ubl domain superimposed with other TBC-B Ubl structures. Structures are shown as tubes coloured by structure: yellow, *Tb*TBC-B; cyan, *C. elegans*; magenta, *D. melanogaster*; slate, *M. musculus*. Affinity tags, where present, have been omitted from the models. The N-terminus is at the top, and the C-terminus points to the bottom. (B) The *Tb*TBC-B CAP-Gly domain superimposed on other TBC-B CAP-Gly domains, in a similar way as in (A): yellow, *Tb*TBC-B; cyan, *C. elegans*; pink, *M. musculus*. The N-terminus is on the left side, and the C-terminus is on the right.

The Ubl module, in terms of amino acid sequence, is the more variable domain of TBC-B (Fig. 3) as compared with homologues. For example, the Ubl and CAP-Gly domains of *T. brucei* and mouse TBC-Bs share ∼ 30% and 55% sequence identity, respectively. Although remarkably similar in structure to ubiquitin (rmsd of 0.83 Å over 41 Cα atoms with PDB code 1UBQ), the *Tb*TBC-B Ubl domain shares only 10% sequence identity. Ubiquitin contains seven lysines (residues 6, 11, 27, 29, 33, 48, and 63) that are targets for covalent modification 15–16. Intriguingly, and despite only low sequence conservation, the Ubl domain of *Tb*TBC-B contains two lysines (Lys34 and Lys62), which correspond to Lys27 and Lys48 of ubiquitin, and a structural overlay reveals strong structural similarity in terms of the positioning of these residues (Fig. 4). The Ubl domains of known structure overlap at a similar level (Fig. 5A).

### The CAP-Gly domain

Crystals of the CAP-Gly domain of *Tb*TBC-B were obtained from a construct producing the 81 C-terminal amino acids. The peptide eluted from the size exclusion column as a single species of ∼ 11 kDa, indicating a monomer in solution. The crystals are orthorhombic with space group *P*2_1_2_1_2_1_, with two polypeptides, labelled A and B, in the asymmetric unit. The rmsd observed after superposition of 79 Cα positions of molecule A on molecule B was 0.86 Å. No restraints were imposed on noncrystallographic symmetry during refinement, so this indicates that the molecules are highly similar. The numbering of residues and secondary structure elements in the C-terminal domain is carried on from the Ubl domain.

Although the sequence derived from the genome assigns residue 223 as aspartate, the cloned gene encodes a glutamate at this position, an observation confirmed by the structural analysis. This conservative difference could be an artefact of the cloning process, or, more likely, could result from a natural variation in the strain that from which genomic DNA was obtained. We note that the *T. brucei gambiense* gene sequence for TBC-B (http://www.genedb.org/) encodes a glutamate in this position.

The *Tb*TBC-B CAP-Gly domain is a small globular structure with a similar fold to other CAP-Gly domains, and is an important module for the recognition and binding of the C-terminal tail of α-tubulin 17–18. Structural comparison reveals that *Tb*TBC-B matches closely to other CAP-Gly domains (Fig. 5B). The *C. elegans* TBC-B CAP-Gly domain, which shares ∼ 50% sequence identity, is the closest structural relative, with an rmsd of 1.4 Å for 95 Cα atoms. The fold is dominated by five β-strands, β5–β9, forming a consecutive twisted antiparallel sheet with β9 returning to lie in an antiparallel fashion at the other side of β5. The side of strand β8 forms the floor of a solvent-exposed, primarily basic groove, which is flanked by the two extended loops, linking β6 and β7, and β7 and β8. NMR studies have revealed that the α-tubulin C-terminal tail peptide binds to this groove in the CAP-Gly domain of human CAP-Gly domain containing linker protein 170 (CLIP-170) 19. Several glycines (residues 170, 188, 195, 200, 215, and 225), which are responsible for the name of this domain, are highly conserved and contribute to the fold of the domain 17. Residues around the peptide-binding groove are also highly conserved, Phe216, Leu180, Trp185, Val201 and Phe207 forming a hydrophobic core that helps to stabilize the floor of the groove.

**Fig 6:**
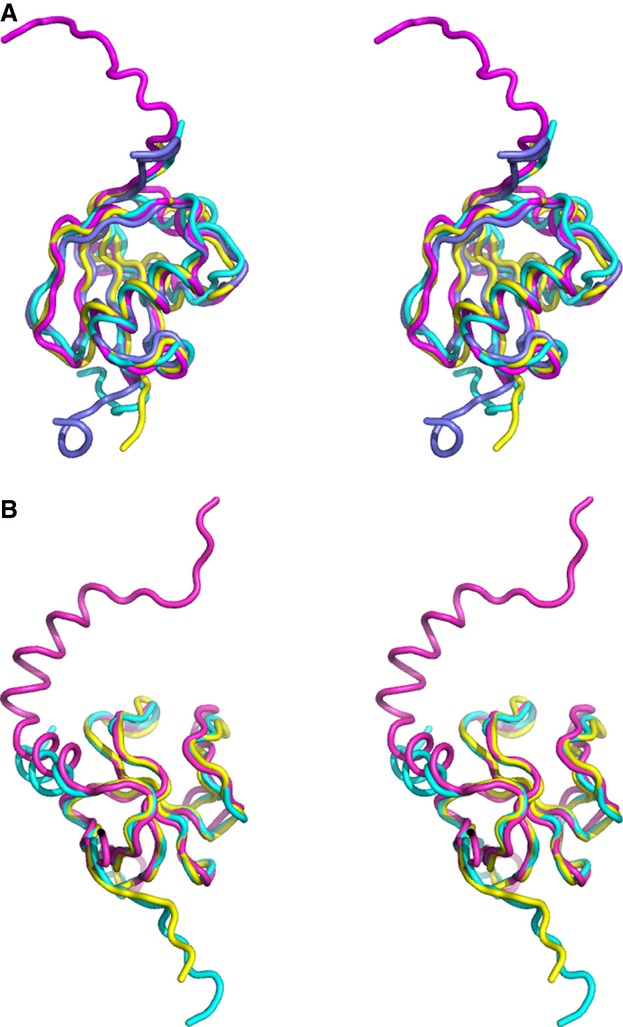
The Asp191 and Arg159 salt bridge in the CAP-Gly domain. The main α trace is shown as grey sticks. Hydrogen bonds are shown as black dashed lines. Conserved residues are shown as sticks coloured by atom type: C, grey; N, blue; O, red, S, yellow.

On the other side of the domain from the peptide-binding groove, there are two salt bridges formed by Arg159 with Asp191, and Arg172 with Glu189, which link β5 with β7, and β6 with β7, respectively. In addition, hydrogen bonds between Arg159 and the backbone oxygen of Phe227 link β5 to the C-terminal segment of β9 (Fig. 6).

CAP-Gly domains, including those of TBC-B, possess a highly conserved pentapeptide tubulin-binding motif with a sequence that is almost always GKNDG 19–20. This motif is placed on one of the loops that flank the peptide-binding groove. NMR studies of CLIP-170 indicate that the asparagine at position 3 can participate in hydrogen bond formation with the C-terminal tail of peptides ending with the sequence EE[Y/F]. This is the sequence found at the C-terminus of α-tubulin and several MT tip-binding proteins. Occasionally, the asparagine in the tubulin-binding motif is replaced by a histidine, a conservative change that does not affect peptide binding. However, mutating either the lysine or the asparagine to alanine has a deleterious effect on binding 20. The *Tb*TBC-B CAP-Gly domain has a different sequence in this tubulin-binding motif at residues 195–199, GKGDG. Glycine rather than asparagine occupies the third position, and this nonconservative change precludes the formation of a side chain hydrogen bond with the terminal residue of an EE[Y/F] peptide.

**Fig 7:**
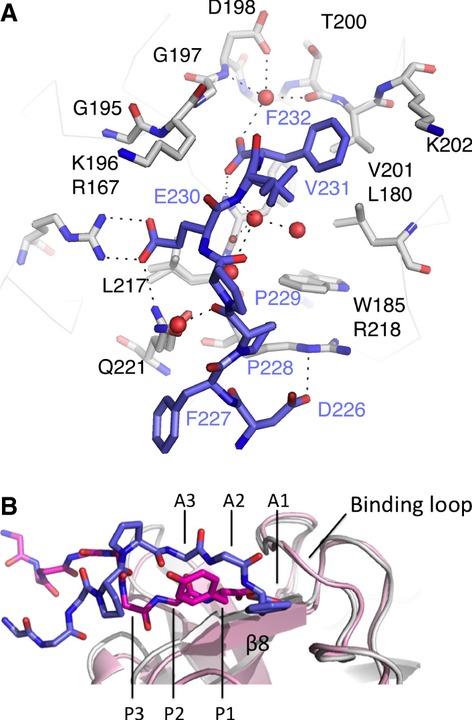
(A) Chain A C-terminus interacting with the peptide-binding groove of a symmetry-related molecule, chain B. The chain B main chain is shown in grey ribbon style, and residues that surround the C-terminal tail are shown as sticks coloured according to atom type: C, grey; N, blue; O, red. Chain A is shown as sticks coloured according to atom type: C, slate blue; O, red. Hydrogen bonds are shown as black dashed lines. Residue numbers for chain A are shown in cyan. L217 and Q221 (chain B) and V231 (chain A) are shown with two side chain rotamer conformations. G199 and F216 are not labelled. (B) Relative positions of the C-terminus peptides in *M. musculus* CAP-Gly domain II of CLIP-170 structure (pale pink cartoon; peptide carbon atoms are in magenta) and *Tb*TBC-B (chain B as a white cartoon; chain A carbon atoms are in slate). For both peptides: O, red; N, dark blue. Amino acids, for clarity, are shown as backbones, except for proline and the C-terminus residues. Peptides are labelled 1–3 from the C-terminus: A for *Tb*TBC-B chain A; P for *M. musculus* polypeptide.

No crystal structures have been solved with an α-tubulin tail-like ligand bound to TBC-B, and, despite our efforts, it was not possible to cocrystallize the *Tb*TBC-B CAP-Gly domain with peptides either. However, and fortuitously, in our structure the C-terminus of chain A forms intermolecular contacts with the basic groove of a symmetry-related chain B (Fig. 7A). The symmetry operation is (− *x* + 1/2, − *y*, *z* + 1/2). The loss of these intermolecular contacts may help to explain why constructs in which the C-terminus had been truncated did not crystallize. The C-terminus of *Tb*TBC-B has the sequence EVF, which is similar to the α-tubulin tail EE[Y/F] motif, and so these intermolecular interactions can be taken to mimic the association with a relevant binding partner. The terminal residue of chain A, Phe232, anchors the peptide in the groove with a combination of hydrophobic and hydrophilic interactions. The aromatic side chain interacts with a hydrophobic patch consisting of the chain B residues Leu180, Val201, and Phe216. The carboxylate of this terminal residue forms a hydrogen bond with the main chain amide of Phe216, and participates in a water-mediated hydrogen-bonding network with Asp198 and Thr200. The C-terminal peptide then arches away from β8, trailing back to Glu230, which forms a salt bridge with Arg167. Another residue, Gln221, interacts with Glu230, as well as contributing to a water-mediated interaction with the backbone of Pro228. Gln221 is conserved in the trypanosomatid TBC-B sequences, but is highly variable in other species. The final interaction that chain A undergoes as it exits the groove is a salt bridge between Asp226 and Arg218.

The NMR structure of the CAP-Gly domain of human CLIP-170 complexed with an α-tubulin tail peptide provides an example for comparative purposes 20. The domains share ∼ 50% identity and are structurally well conserved, with an rmsd of 1.06 Å over 50 Cα atoms. In the CLIP-170 α-tubulin tail peptide complex, the peptide is positioned closer to the β8 strand (Fig. 7B).

The Gly/Asp difference at position 4 in the tubulin-binding loop, discussed earlier, allows the C-terminus of chain A to bind further along the groove than the α-tubulin tail peptide in the CLIP-170 structure. This causes a relative shift in the position of the Val231 side chain, which is directed out of the groove, and undergoes no interactions with the CAP-Gly domain. The third residue from the end of chain A, Glu230 (A3 in Fig. 7), is in the same position as the second residue from the end in the α-tubulin tail peptide, Glu450 (P2 in Fig. 7). This may explain why the sequence EVF can bind, contrary to the conclusion that EE[Y/F] is the essential recognition sequence 18–19. In an attempt to further investigate CAP-Gly peptide interactions, we carried out isothermal titration calorimetry (ITC) with the hexapeptide EDVEEY, which represents the C-terminal residues of *T. brucei* α-tubulin. A range of concentrations of the protein domain and the peptide were tested, but no heat changes were observed (data not shown).

The peptide-binding groove on chain A is occupied by two formate molecules, derived from the crystallization mixture, which bind in similar fashion to the negatively charged moieties of the C-terminal peptide just discussed (data not shown). One formate interacts with Arg167 and the other with Gln221.

### Unique features of Trypanosoma TBC-B

CAP-Gly domains recognize the highly conserved α-tubulin C-terminal tail with the sequence EE[Y/F] 18. In *T. cruzi* and *Trypanosoma vivax* α-tubulin, this sequence is matched exactly. In *T. brucei gambiense* and *T. brucei* 427, the sequence is EMF, and, as just described, we present structural data to show that the sequence EVF can also bind to a CAP-Gly domain. Our structure of *Tb*TBC-B indicates that the penultimate residue, whether glutamate or methionine, is probably directed out of the peptide-binding groove, and, with no direct interactions involving the side chain, the identity would appear to be less important for binding.

Both TBC-B and TBC-E retain highly conserved CAP-Gly domains across numerous different species, and this probably reflects important roles in tubulin biology 20. The CAP-Gly domain of TBC-E also usually contains the GKNDG tubulin-binding motif. The closest homologue to human TBC-E in *T. brucei* is *Tb*927.3.2680, a 530-residue protein with ∼ 25% sequence identity. The protozoan protein contains a leucine-rich repeat segment and a Ubl domain but, surprisingly, lacks a CAP-Gly domain. In fission yeast, the TBC-E homologue Alp21 also lacks a CAP-Gly domain, but is indispensible for maintaining α-tubulin levels, MT integrity, and cell survival 21–22. In contrast, mammals possess a TBC-E paralogue, which lacks the CAP-Gly domain. This protein, known as E-like, with ∼ 30% sequence identity with TBC-E, cannot compensate for loss of TBC-E, and, instead of being involved in tubulin biogenesis, is implicated in degradation 23. This difference is perhaps a legacy of a more complex and highly regulated tubulin biology in higher eukaryotes, and suggests that a degree of care is required when considering different model systems.

We could not identify any other CAP-Gly domains encoded by the *T. brucei* genome, even in proteins that usually contain such modules, e.g. kinesins. Perhaps, in trypanosomatids, TBC-B is sufficient to provide this interaction with the α-tubulin tail, or other modules, yet to be discovered, may compensate for this function.

### The missing structural information

As it was not possible to obtain crystallographic data on the linker region between the globular domains (residues 89–153), the CD spectrum of full-length TBC-B was analysed. The spectra indicated a low α-helical content of ∼ 10% and a β-strand content of ∼ 35%. The α-helical content of the Ubl domain itself is ∼ 8% of the overall structure, and, together with the prediction of a short helical segment between residues 116 and 123, ∼ 4% of the sequence, there is excellent agreement with the CD data. Residues with a β-strand conformation constitute nearly 30% of the full-length structure, which is also in good agreement with the spectroscopic data. Both the CD results and the structural analyses also agreed well with the predicted secondary structure, and overall indicate a two-domain structure with a flexible linker.

### Functional implications and concluding remarks

Structures of the Ubl and CAP-Gly domains of *Tb*TBC-B, the first such protist structures, were determined to 2.3-Å and 1.6-Å resolution, respectively. It was not possible to solve the full-length structure, as the crystals did not diffract sufficiently well, and attempts to extend structural information into the region linking the two domains were also unsuccessful. It was determined that the missing linker region is mostly unstructured, with only a short segment of α-helix being noted, and potentially flexible.

The fold of the Ubl domain is highly conserved, despite low sequence identity within TBC-B proteins and, indeed, ubiquitin itself. A striking similarity is observed in the spatial location of two functionally important lysines in ubiquitin, and strongly supports a biological function for the Ubl domain, and by implication for TBC-B. Through reversible post-translational modification, TBC-B can influence aspects of tubulin biology in a proteasome-dependent manner. Such a conclusion is consistent with other studies. Yeast two-hybrid studies suggest that proteasome-dependent degradation of human TBC-B is driven by gigaxonin binding to the Ubl domain 24. In addition, *Saccharomyces cerevisiae* TBC-E interacts with the ubiquitin receptor Rpn10 via the Ubl domain, and subsequently with a ubiquitin ligase complex, providing a route to protein degradation 10. We note also that Lys34, but not Lys62, of *Tb*TBC-B is conserved in the Ubl domains of TBC-E (data not shown).

In the final stages of MT assembly, α-tubulin and β-tubulin form a heterodimer with the encouragement of the TBC proteins. A balance has to be struck between the availability of free tubulin, heterodimer assembly, and release of MT structures. This process involves a number of highly abundant proteins, and the presence of Ubl domains offers a means whereby protein folding and the population of complex assemblies can be regulated by protein degradation or recycling. The use of a Ubl protein rather than ubiquitin itself may provide specificity with regard to this aspect of tubulin biology. Cognate activating enzymes might then contribute to the regulation of levels of free tubulin heterodimers, tubulin polymerization, and MT catastrophe, or to avoid miscommunication during a stress response. It will therefore be of great interest to now identify, from a plethora of candidates, the specific ligases and proteases that might participate in the control of MT disassembly/assembly as opposed to other biological functions.

The molecular packing in the crystal structure of the CAP-Gly domain places the C-terminal tail of one CAP-Gly domain in the peptide-binding groove of a symmetry-related molecule, and allows examination of interactions in this binding site. The binding differs slightly from that observed in a CLIP-170 CAP-Gly domain bound to an α-tubulin tail peptide, owing to a difference in the β7–β8 tubulin-binding loop of *Tb*TBC-B. A glycine replaces asparagine at position 3 of the normally conserved GKNDG sequence, and allows the C-terminus of chain A to bind further down in the peptide-binding groove. This appears to be a trypanosomatid-specific feature.

Tubulin and MT assembly are subject to C-terminal post-translational modifications, which provide important tracking positions for a range of MT-binding partners 25. For example, the C-terminal residue of α-tubulin is a tyrosine that can be removed and then replaced, and adjacent glutamates can be polyglutamylated or, in some species, polyglycylated. The consensus sequence for recognition by CAP-Gly domains has been deduced as EE[Y/F], and proteolytic removal of the tyrosine prevents such recognition of α-tubulin 25. This provides a feature that might be used to regulate subpopulations of MTs. A plausible function of the CAP-Gly domain may therefore be to bind then block post-translational modifications of the C-terminal tyrosine, as the complex with α-tubulin appears to form even while it is still bound to CCT 8, i.e. while the tyrosine is still present.

We were unable to observe any binding of the CAP-Gly domain with a hexapeptide representing the terminal residues of *T. brucei* α-tubulin by using ITC. According to previous work, the presence of TBC-E might be required for TBC-B to form a stable complex with α-tubulin 6. Further experiments would be required to inform hypotheses involving the need for partner proteins and/or post-translational modifications that allow TBC-B to contribute to MT assembly or disassembly.

## Experimental procedures

### Protein production and purification

The gene encoding full-length *Tb*TBC-B was amplified from genomic DNA (strain 927) with 5′-CATATGTCCGTTGTTAAAGTATCGC-3′ and 5′-CTCGAGTTAAAACACCTCCGGGGGAAAGTC-3′ as forward and reverse primers, respectively (ThermoFisher Scientific, Waltham, MA, USA). The restriction enzyme sites for *Nde*I and *Xho*I are in bold. The PCR product was ligated into pCR2.1-TOPO with the TOPO TA Cloning Kit (Invitrogen, Carlsbad, CA, USA), and then cloned into a modified pET15b (Novagen, Madison, WI, USA) expression vector, which adds an N-terminal His_6_ tag and a tobacco etch virus (TEV) protease cleavage site to the product. Sequencing confirmed the identity of the construct, and the vector was heat-shock-transformed into *E*. *coli* Rosetta (DE3) pLysS cells for expression. DNA encoding the CAP-Gly domain, residues 157–222, was amplified from the vector containing the *Tb*TBC-B gene with 5′-CATATGGCAGAGACAATACATGTGGGGG-3′ and 5′-CTCGAGTTAAAACACCTCCGGGGGAAAGTC-3′ as forward and reverse primers, respectively (ThermoFisher Scientific). The gene fragment was cloned into the modified pET15b vector as described above, and transformed into *E. coli* Rosetta (DE3) pLysS cells for protein production.

Similar protocols were applied to purify full-length *Tb*TBC-B and the CAP-Gly domain. Typically, cells were grown at 37 °C in 1 L of LB medium supplemented with carbenicillin (50 μg·L^−1^) and chloramphenicol (25 μg·L^−1^). Gene expression was induced, at a *D*_600 nm_ of 0.6, by addition of 1 mm isopropyl thio-β-d-galactoside. The culture was incubated for a further 16 h at 22 °C, and cells were then harvested by centrifugation at 3500 ***g*** for 30 min at 4 °C. The cells were resuspended in a lysis buffer (50 mm Tris/HCl, pH 7.5, 250 mm NaCl, 25 mm imidazole) containing DNase I and EDTA-free protease inhibitors (Roche, Basel, Switzerland), and then lysed with a French press at 16000 p.s.i. The resultant lysate was centrifuged at 35 000 ***g*** for 30 min at 4 °C, and the supernatant was loaded onto a pre-equilibrated HisTrap HP 5-mL column (GE Healthcare, Milwaukee, WI, USA) precharged with Ni^2+^. A linear gradient of 25 mm to 1 m imidazole was applied to elute the proteins, and the derived fractions were analysed by SDS/PAGE. Samples were pooled and incubated with His-tagged TEV protease at 30 °C for 3 h, and then dialyzed into 50 mm Tris/HCl (pH 7.5) and 250 mm NaCl. The sample was loaded onto a HisTrap HP 5-mL column to remove the His-tagged TEV protease, uncleaved product, and histidine-rich contaminants. A Superdex 20 026/60 size exclusion column (GE Healthcare) equilibrated with 50 mm Tris/HCl (pH 7.5) and 250 mm NaCl was used to further purify the protein. This column had been calibrated with BioRad Gel Filtration standards. Fractions containing the proteins were pooled and concentrated to 10 mg·L^−1^ with Amicon Ultra devices (Millipore) for subsequent use. The purity and identity of the proteins were confirmed by MALDI-TOF MS. Yields of ∼ 20 mg·L^−1^ cell culture for full-length TBC-B and ∼ 8 mg·L^−1^ cell culture for the CAP-Gly domain were obtained.

A series of constructs encoding five polypeptide fragments, covering residues 91–232, 101–232, 113–232, 128–226, and 143–232, were also produced in an attempt to extend structural knowledge between the domains. These polypeptides proved to be either insoluble or failed to crystallize, and so no further details are provided.

### CD of full-length TbTBC-B

CD spectra were recorded on a Jasco J-810 spectropolarimeter. Far-UV CD spectra were obtained with 0.5 mg·mL^−1^ protein solutions and a 0.02-cm-pathlength quartz cuvette. Five scans were accumulated and averaged with the following parameters: scan rate, 50 nm·min^−1^; response, 0.5 s; and bandwidth, 1 nm. Protein CD spectra were corrected by subtracting the appropriate buffer spectrum and correcting for protein concentration. Protein secondary structure estimates were obtained with the contin procedure 26, available from the dichroweb server 27.

### Analysis of crystals formed following proteolysis

Crystals of the full-length protein did not diffract beyond ∼ 7-Å resolution (data not shown). Limited proteolysis by addition of chymotrypsin to the crystallization drops 28 was therefore tested in the search for ordered crystals. Crystals were observed in a number of conditions, one of which was successfully optimized, as detailed below. These crystals were harvested, washed in the reservoir solution, dissolved in ddH_2_O, and then submitted for MALDI-TOF MS analysis. This gave a molecular mass of 12 069 Da for the polypeptide. The fragment was isolated by SDS/PAGE and then transferred onto a poly(vinylidene difluoride) membrane. The band was then submitted for N-terminal Edman sequencing, and the sequence GHMSVVKV was identified. This corresponds to the N-terminus of *Tb*TBC-B, with the first two residues being remnants of the TEV cleavage site after treatment to remove the His_6_ tag. These data indicated that the Ubl domain had crystallized.

### Isolation of products after chymotrypsin treatment

Full-length *Tb*TBC-B (50 mg) was incubated with chymotrypsin (0.02 mg) overnight, and then dialyzed into 50 mm Tris/HCl (pH 7.5) and 50 mm NaCl. The mixture was loaded onto a HiTrap Q HP 5-mL column, and eluted with a linear gradient of 50 mm to 1 m NaCl. The products of the cleavage eluted as two peaks, and were analysed by SDS/PAGE. Sample 1 eluted at 50 mm NaCl, and sample 2 at 220 mm NaCl. MALDI-TOF MS of sample 1, the Ubl domain, gave a mass of 12 002 Da, and MALDI-TOF MS of sample 2 gave a mass of 12 116 Da. Both protein fragment samples were concentrated to 5 mg·mL^−1^.

### Selenomethionine (SeMet) derivative of the Ubl domain

A SeMet-substituted Ubl domain was obtained from material generated as described above, and with incorporation achieved following metabolic inhibition 29. Purification of the protein yielded ∼ 2 mg·L^−1^ of cell culture. Analysis by MALDI-TOF MS indicated full incorporation of four SeMet residues.

### Crystallization and data collection

Initial crystallization conditions were established by sitting drop vapour diffusion with a Phoenix Liquid Handling System (Rigaku; Art Robins Instruments, Mountain View, CA, USA) and commercially available screens. Several conditions were optimized for hanging drop vapour diffusion, with drops of 1 μL of protein solution and 1 μL of reservoir.

Rectangular crystals (dimensions 0.3 × 0.1 × 0.1 mm) of the Ubl domain, which had been isolated after proteolysis of the full-length protein, were obtained with a reservoir of 0.1 m Hepes (pH 7.5), 1% poly(ethylene glycol) 400, and 2 m ammonium sulfate, and a protein concentration of 2.5 mg·mL^−1^. Crystals of the CAP-Gly domain, derived from expression of a gene fragment, were rod-like, with dimensions of 1 × 0.05 × 0.05 mm, and were obtained with reservoir conditions of 0.2 m potassium formate, 30% poly(ethylene glycol) 3350 and a 7.5 mg·mL^−1^ protein solution in 50 mm Tris/HCl (pH 7.5) and 250 mm NaBr.

Crystals were transferred to a solution containing a mixture of their reservoir solution and 40% poly(ethylene glycol) 400 for ∼ 15 s before being cooled to −173 °C in a stream of nitrogen. The crystals were characterized in-house with a Micromax HF007 copper-rotating anode X-ray generator equipped with an R-axis IV^++^ dual image plate detector. Subsequently, data were collected with ADSC charge-coupled device detectors at the Diamond light source beamline I03 for the Ubl domain and at beamline I02 for the CAP-Gly domain.

Data from the Ubl domain crystal were processed with xds 30 and scala 31. Initial phases were obtained with SeMet single-wavelength anomalous dispersion methods 14, with data measured at the experimentally determined *f*′ maximum wavelength (*λ* = 0.98 Å) in the ccp4 pipeline 32 with crank 33. A figure-of-merit of 0.23 was obtained from bp3 34, and this increased to 0.67 after density modification and solvent flattening in solomon 35. This phase set was used for initial model building with buccaneer 36.

Data from the CAP-Gly domain crystal were scaled and processed with imosflm 37 and scala 31. The phyre server 38 identified the NMR structure of the *M. musculus* CAP-Gly domain of TBC-B (PDB code 1WHG), sharing a sequence identity of ∼ 55%, as a suitable model for molecular replacement. A poly-Ala model was prepared with chainsaw 39, and the positions of two molecules were then identified with phaser 40. Rigid body refinement gave an initial *R*-factor of 49% to 1.6-Å resolution.

Both structures were refined with rounds of map inspection and model manipulation with coot 41 and refinement calculations with refmac 42. When the protein models were nearly complete, waters and ligands (ethylene glycol in the Ubl domain and formate in the CAP-Gly domain) were added, together with multiple conformers for several side chains. The geometric quality of the models was assessed with molprobity 43. Figures were created with pymol 44, and solvent accessibility analyses were performed with areaimol 32. Crystallographic statistics are presented in Table 1. Coordinates and structure factors are deposited with the PDB under accession codes 4B6W for the Ubl domain and 4B6M for the Cap-Gly domain.

**Table 1:** *Tb*TBC-B domain crystallographic statistics

	Ubl domain	CAP-Gly domain
Space group	*P*4_1_2_1_2	*P*2_1_2_1_2_1_
Unit cell lengths (Å)	50.9, 50.9, 77.2	32.7, 55.7, 80.4
Resolution range (Å)	36.00–2.35	40.18–1.59
Completeness (%)	100 (99.8)^a^	100 (99.97)
*<I/σ*(*I*)*>*	15.4 (3.3)	28.2 (9.6)
Reflections measured/unique	58 686/4631	282 648/20 384
Redundancy	12.7 (9.4)	13.9 (14.1)
Anomalous redundancy:	7.1 (5.0)	NA
*R*_merge_^b^	0.12 (0.64)	0.07 (0.28)
*R*_work_^c^/*R*_tree_^d^	0.16/0.21	0.20/0.24
Protein residues (chain A/B)	89	79/78
Ligands (number)	Ethylene glycol (1)	Formate (6)
Rmsd from ideal geometry		
Bond lengths (Å)/angles (^o^)	0.015, 1.53	0.01, 1.44
Thermal parameters (*B*, Å^2^)		
Wilson *B*	42.6	14.6
Mean *B* (all atoms)	20.1	14.8
Protein atoms (chainA/B)	18.0	12.3/12.5
Ligands	58.9	21.0
Water molecules	42.4	24.8
Ramachandran plot		
Favored/allowed (%)	97.6/2.4	98/2

NA, not analysed.

a Values in parentheses refer to the highest-resolution bin.

b *R*_merge_ = ∑*h∑i||*(*h,i*) − <*I*(*h*)> ∑*h∑I* I(*h,i*).

c *R*_work_ = ∑*hkl*||*F*_*o*_|–|*F*_*c*_||/∑*|F*_*o*_*|*, where *F*_*o*_ is the observed structure factor amplitude and *F*_*c*_ is the structure-factor amplitude calculated from the model.

d *R*_free_ is the same as *R*_work_ except that it was only calculated using a subset, 5%, of the data that are not included in any refinement calculations.

### Bioinformatic analyses

Secondary structure and disorder predictions were obtained from phyre 38 and psipred 45. Structural comparisons and homologues were identified with dali 46. Conserved residues within the CAP-Gly domain were identified with consurf 47. The UniProt reference cluster 90 (UNIREF90) 48 database was searched, and sequences were aligned with mafft 49. Overall, 642 sequences were identified with psi blast 50, on the basis of sequence identity in the range 35–95%, 417 of which were unique. A subset of sequences, 150, the default value in consurf, with identity > 50% were used for comparisons. However, there were not enough homologues with identities of > 30% of the Ubl domain for consurf to be used, so 31 sequences with identities in the range 31–38%, were aligned by the use of muscle 51, and residues with > 75% conservation were annotated.

### ITC details

ITC experiments were performed at 25 °C with a VP-ITC calorimeter (MicroCal, Northampton, MA, USA). The 1.4-mL sample cell and 300-μL syringe were filled with TBC-B CAP-Gly domain and peptide EDVEEY in a buffer of 50 mm Tris/HCl (pH 7.5) and 250 mm NaCl. Typically, 8 μL of peptide was injected from the stirred syringe (305 r.p.m.) 30 times into the sample cell. The peptides were dissolved in the minimum amount of the buffer, and then diluted to the appropriate concentration just before use. Four experiments were carried out with CAP-Gly/hexapeptide concentrations of 1 : 10, 10 : 100, 7 : 500 and 45 : 500 μL. No binding events were observed.

## Abbreviations

CAP-Gly: cytoskeleton-associated protein with glycine-rich segment
CCT: chaperonin containing T-complex polypeptide 1
CLIP-170: cytoskeleton-associated protein with glycine-rich segment domain containing linker protein 170
ITC: isothermal titration calorimetry
MT: microtubule
PDB: Protein Data Bank
SeMet: selenomethionine
TBC: tubulin-binding cofactor
TbTBC-B: *Trypanosoma brucei* tubulin-binding cofactor B
TEV: tobacco etch virus
Ubl: ubiquitin-like
